# Hydrothermal Carbonization of Pruned Persimmon Tree Branches: Optimization of Process Conditions for Enhanced Energy Recovery

**DOI:** 10.3390/ma18153425

**Published:** 2025-07-22

**Authors:** Hirotaka Maeda, Yuta Ueda

**Affiliations:** 1 Advanced Ceramics Program, Graduate School of Engineering, Nagoya Institute of Technology, Gokiso-cho, Showa-ku, Nagoya 466-8555, Japan; 2 Creative Engineering Program, Graduate School of Engineering, Nagoya Institute of Technology, Gokiso-cho, Showa-ku, Nagoya 466-8555, Japan

**Keywords:** hydrothermal carbonization, persimmon pruning branches, agricultural waste management, energy resource

## Abstract

Pruned branches from persimmon trees are a largely untapped agricultural waste resource. This study explores the conversion of these branches into an enhanced fuel source through hydrothermal carbonization. The branches were subjected to hydrothermal treatment under various conditions to identify the optimal parameters. Higher temperatures and longer treatment durations increased the carbon content to 69.2% and reduced the oxygen content to 20.4%. A Van Krevelen diagram showed that dehydration was the primary reaction, with decarboxylation occurring at 250 °C. The energy value increased from 18.2 MJ/kg for raw branches to 28.5 MJ/kg under the optimal conditions, indicating a 57% improvement. These findings demonstrate that hydrothermal carbonization effectively utilizes persimmon pruning waste, offering a sustainable method for converting biomass into energy and aiding agricultural waste management.

## 1. Introduction

The effective utilization of biomass to produce valuable commodities such as liquid fuels and chemicals is facilitated by various processes, including thermochemical and chemical conversion. This strategy supports the establishment of a sustainable society and addresses the pressing need for renewable resources and waste reduction in the food industry. Recently, numerous studies have used non-edible agricultural wastes, such as rice husk, as starting materials to prepare functional materials for environmental and fuel applications [1,2,3]. Pruning residues from roadsides and fruit trees are significant, yet underutilized, resources in many regions. Although initiatives have been undertaken to repurpose these materials as fuels or compost [4,5], their overall utilization remains suboptimal. This underutilization results in a substantial amount of organic waste, which is frequently disposed of through incineration or open-burning practices. These disposal methods not only squander potential resources, but also contribute to global environmental pollution.

In 2023, China, the Republic of Korea, and Japan were the leading producers of persimmons according to the FAOSTAT statistical database of the Food and Agriculture Organization of the United Nations. This suggests the generation of significant amounts of pruning waste. These pruned persimmon branches are categorized as lignocellulosic biomass, consisting of approximately 35% cellulose, 15% hemicellulose, and 28% lignin, which is a higher lignin content than that found in other orchards, such as pears and apples [6]. Bioconversion of pruned persimmon branches produces lignin nanoparticles and cellulosic ethanol [7]. Activated carbons and biochars derived from pruned persimmon branches have a demonstrated potential for use in battery capacitors, energy generation, and environmental remediation [8,9,10,11].

The higher heating value (HHV) of biomass is positively correlated with its lignin content [12]. Pruned branches of persimmon trees have been found to possess a slightly higher heating value than other orchard branches [6], indicating their potential as a biomass source for alternative energy production. Lignocellulosic biomass undergoes carbonization via pyrolysis or hydrothermal treatment to enhance its energy conversion efficiency. Hydrothermal treatment is particularly beneficial for agricultural biomass with high moisture content. When upgrading the waste wood of Eucommia ulmoides Oliver into solid fuels, hydrothermally treated samples exhibit a higher fuel rate than pyrolyzed samples at the same temperature [13]. We believe that hydrothermal treatment is suitable for converting biomass into energy. To the best of our knowledge, limited information is available regarding the conversion of pruned persimmon branches into energy through hydrothermal treatment. The conditions under which hydrothermal carbonization of biomass is conducted significantly influence the properties of the resulting products [14,15]. This necessitates the optimization of the hydrothermal conditions for pruning persimmon tree branches. In this study, we explored the potential of pruned persimmon branches as a fuel source through hydrothermal conversion under various conditions.

## 2. Materials and Methods

Branches of a sweet Japanese persimmon tree collected from Gifu Prefecture were pruned. The collected branches were stored at room temperature without humidity control to replicate the environmental conditions under which such materials are typically maintained. These branches were dried at 80 °C for four nights before being pulverized. The dried persimmon tree pruning branches were then sieved using a 300 μm mesh, with the particle distribution curve shown in Supplementary Material Figure S1. A slurry was prepared by adding adequate amounts of persimmon branch powder to 10 mL of distilled water in a 50 mL Teflon container. Hydrothermal treatment was conducted in a Teflon-lined stainless-steel autoclave using a roller oven operating at approximately 45 rpm. The variables considered in this process included temperature (180–250 °C), duration (2–40 h), and solid/liquid ratio (2.5, 5, 10), as listed in Table 1. Following hydrothermal treatment, the samples were washed with distilled water, separated by filtration, and dried overnight at 100 °C. The resulting samples were stored in a vacuum desiccator until further analysis.

The crystallinity of the samples was assessed using X-ray diffraction (XRD; Malvern Panalytical, X’pert, Malvern, UK) with CuKα radiation at 45 kV and 40 mA, employing a scanning rate of 0.1 degree/s. To determine the crystallinity index (CI) of cellulose from the XRD pattern, the height ratio between the intensity of the crystalline peak (I_002_–I_AM_) and the total intensity (I_002_) was employed [16]. Background signals from the XRD patterns were subtracted using Origin Pro 2025 (OriginLab, Northampton, MA, USA). I_002_ and I_AM_ represent the intensities of the crystalline peak at approximately 21.5° and the amorphous phase peak at approximately 18°, respectively. Fourier transform infrared (FT-IR; JASCO, FT/IR-6100, Tokyo, Japan) spectroscopy was used to analyze the samples using the KBr method. An elemental analyzer (Elementar, Vario EL cube, Langenselbold, Germany) was used to determine the carbon, hydrogen, nitrogen, and sulfur content of the samples. The ash content of each sample was determined by measuring the weight loss after heating at 815 °C for 1 h. The heating program involved raising the temperature to 500 °C over 60 min, followed by an increase to 800 °C in 45 min, as specified in JISM8812:2006. The oxygen content was calculated using Equation (1) [17]:

O = 100 − (C + H + N + S + Ash)(1)

To evaluate the combustion properties of the samples, their HHV were calculated using Equation (2) [18]:

HHV = 0.3491C + 1.1783H + 0.1005S − 0.1034O − 0.015N + 0.0221Ash(2)

## 3. Results and Discussion

**Figure 1:**
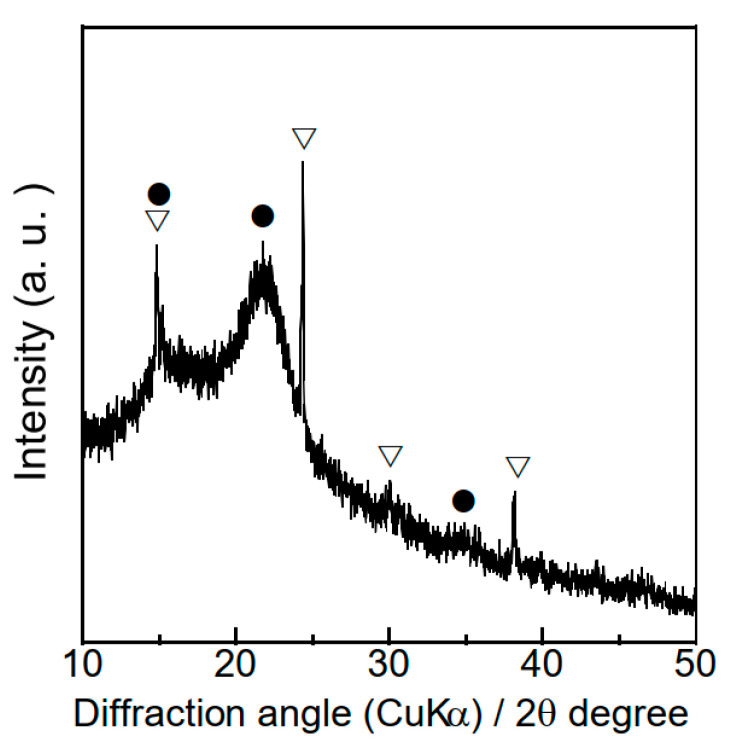
XRD pattern of the pruned persimmon tree branch powders. (▽) calcium oxalate, (●) crystalline cellulose.

Figure 1 shows the XRD patterns of the persimmon branch powders, which contained crystalline cellulose and calcium oxalate. The CI of the cellulose was 58%. Elemental analysis revealed that pruned persimmon tree branch powders contained 44.9 wt% carbon, 45.2 wt% oxygen, 6.2 wt% hydrogen, and 1.1 wt% nitrogen. Although ambient storage was adopted in this study to simulate practical conditions, uncontrolled humidity can lead to material degradation over time. Therefore, the development of appropriate storage strategies such as humidity-controlled or sheltered storage areas would be beneficial for preserving feedstock quality for long-term use.

**Figure 2:**
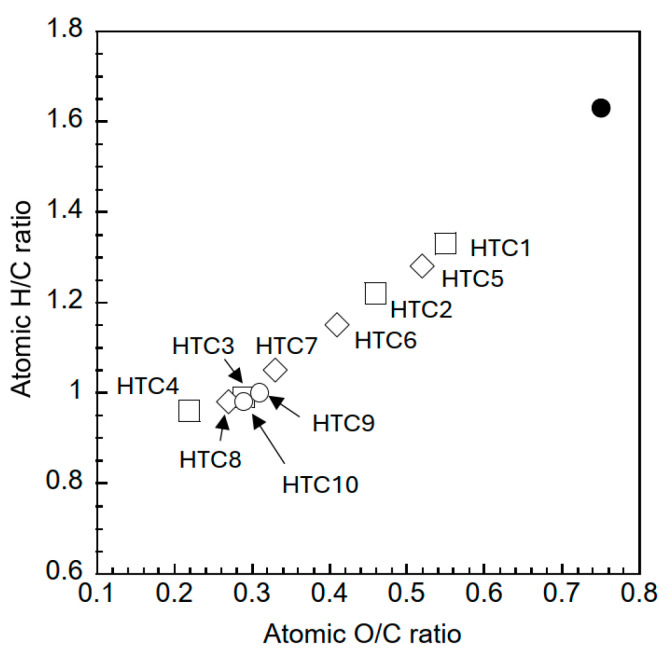
Van Krevelen diagram of the samples. (●) pruned persimmon tree branch; (□) HTC1-4, temperature effect; (◇) HTC5-8, time effect; (◯) HTC9,10, solid/liquid effect.

The elemental compositions and solid yields of the samples were analyzed using two separately prepared samples, and the resulting averages are presented in Table 2. An increase in temperature and duration resulted in an elevated carbon content and a reduction in the oxygen content within the samples. The hydrogen, nitrogen, and sulfur contents had minimal influence on the hydrothermal conditions. The yield was dependent on the temperature and duration of hydrothermal treatment. The solid/liquid ratio increased the yield, although the carbon content of the samples remained relatively constant. The carbon balance, calculated from the solid yield and carbon content before and after hydrothermal treatment, ranged from 53% to 68%, depending on the reaction conditions. The carbon balance tends to depend strongly on the solid yield under hydrothermal conditions. The influence of the hydrothermal conditions on the H/C and O/C atomic ratios of the samples was examined using a Van Krevelen diagram, as depicted in Figure 2. The persimmon tree pruning branches exhibited O/C and H/C ratios of 0.75 and 1.63, respectively. An increase in both temperature and duration led to a reduction in the O/C and H/C ratios of the samples, primarily because of dehydration. An increase in the solid content during hydrothermal treatment is likely to enhance the carbonization reaction, resulting in a reduction in the O/C and H/C ratios of the samples. The temperature and duration under hydrothermal conditions exerted a more pronounced effect on the carbonization process than on the solid/liquid ratio. Hydrothermal treatment at 250 °C facilitated the decarboxylation reaction in addition to the dehydration reaction. Table 3 shows a comparison of the data from the other biomass samples subjected to hydrothermal treatment. Hydrothermal treatments at similar temperatures and durations are expected to result in comparable carbonization and solid yields, regardless of the biomass species. The duration of the hydrothermal treatment significantly influenced the carbonization process at similar temperatures.

**Figure 3:**
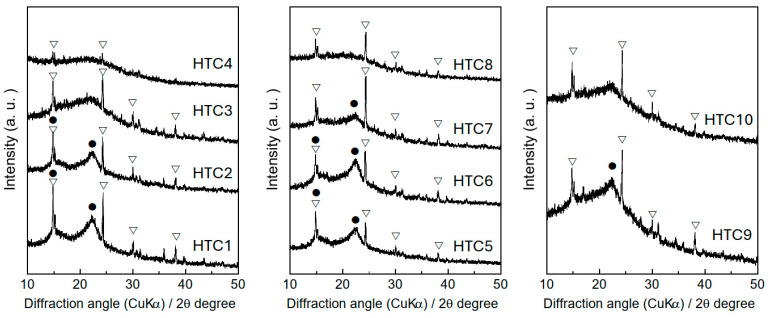
XRD patterns of the samples. (▽) calcium oxalate, (●) crystalline cellulose.

Figure 3 shows the XRD patterns of the samples. Peaks corresponding to calcium oxalate were evident across all the patterns, signifying the persistence of calcium oxalate crystals in the samples, regardless of the hydrothermal conditions. Furthermore, peaks associated with crystalline cellulose were observed in the XRD patterns of HTC1, 2, 5–7, and 9. The CI of cellulose, as shown in Table 2, exhibited a slight increase below 205 °C with a hydrothermal treatment duration of 20 h. However, as the duration extended, the CI gradually decreased at the same temperature. These findings suggest that crystalline cellulose tends to transition to an amorphous phase at temperatures exceeding 230 °C with a hydrothermal duration of 20 h. The solid/liquid ratio under hydrothermal conditions also affects the hydrolysis of crystalline cellulose under experimental conditions. These results suggest that the transformation of cellulose promotes the carbonization of pruned persimmon tree branches.

**Table 3:** Hydrothermal conditions for various biomass types, along with the atomic ratio and yield of the hydrochar.

Biomass	Temperature (°C)	Duration (h)	H/C	O/C	Solid Yield (%)
Wood waste [19]	220	2	1.27	0.48	~65
Cellulose extracted from poplar [20]	230	12	1.00	0.25	~38
Typha australis [14]	233	10	0.90	0.2	~40
Persimmon tree pruning branches (this work)	230	20	0.99	0.29	41.5

**Figure 4:**
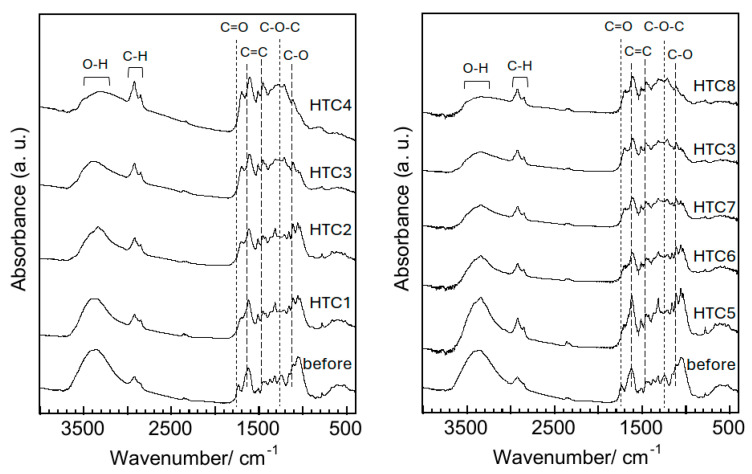
FT-IR spectra of persimmon tree pruning branches before and after hydrothermal treatment.

Figure 4 shows the FT-IR spectra of pruned persimmon tree branches before and after hydrothermal treatment. Initially, the spectrum of the branches before treatment exhibited two distinct bands: one at approximately 1235 cm^−1^ corresponding to the C-O-C bonds of the acetyl groups in hemicellulose [21], and the other at approximately 1730 cm^−1^ associated with the C=O stretching vibration of the acetoxy groups in xylans [22]. These bands were absent from the obtained spectra, even after the application of the lowest temperature and shortest hydrothermal treatment. Increases in temperature and duration tended to reduce the band associated with the O-H stretching vibration between 3300 and 3500 cm^−1^ [23] in the spectra of HTC1-8, implying dehydration of the branches during the hydrothermal treatment. The bands corresponding to aliphatic C-H stretching vibrations at approximately 2850 and 2920 cm^−1^ [24] were evident in all sample spectra, indicating that no demethanation occurred during the treatment. These findings were consistent with the Van Krevelen diagram. Bands attributed to C-O in the methoxy groups in lignin at approximately 1110 cm^−1^ [23], the C=C stretching vibration under aromatic groups in lignin at approximately 1450 cm^−1^ [23], and the C=C stretching vibration of aromatic rings in lignin at approximately 1610 cm^−1^ [25] were consistently observed across all spectra. A band indicating the formation of new materials resulting from condensation reactions between carbohydrate and lignin products is clearly visible at approximately 1705 cm^−1^ [26] in the post-treatment spectra. This suggests that hemicellulose decomposes more rapidly than lignin because of its lower thermal stability, which contributes to the formation of the reaction products. The hydrothermal degradation of lignin and cellulose is most likely achievable at temperatures exceeding 200 °C [27]. While the spectral bands linked to lignin were identified in the sample subjected to hydrothermal treatment at 250 °C, lignin is anticipated to partially degrade, resulting in a decarboxylation reaction as illustrated in the Van Krevelen diagram. The reaction mechanism at 230 °C is speculated to proceed as follows: after 2 h of hydrothermal treatment, the chemical bonds in hemicellulose begin to decompose, whereas cellulose gradually loses its crystallinity over a span of 20 h. By contrast, the chemical bonds in the lignin remained largely intact even after 40 h. HTC9 and HTC10 exhibited nearly identical FT-IR spectra (Supplementary Material Figure S2), indicating that the solid/liquid ratio under hydrothermal conditions had minimal impact on the chemical bonds of the samples.

**Figure 5:**
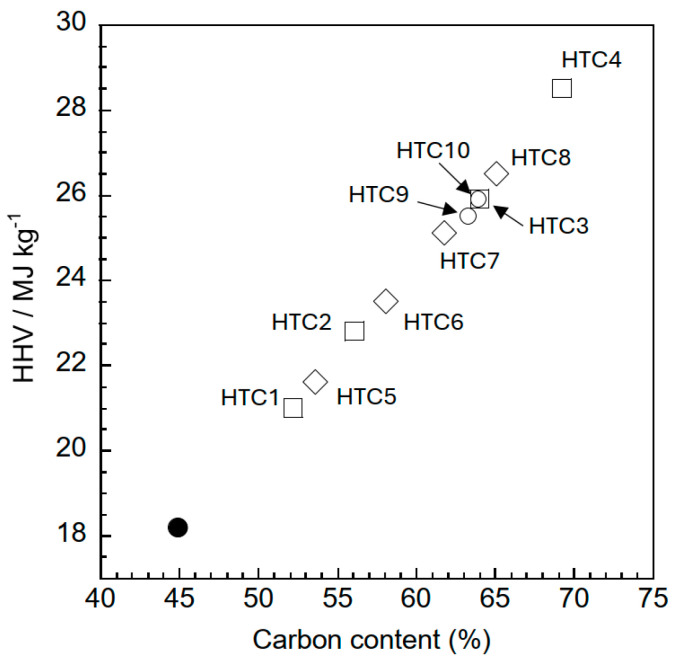
Relationship between the HHV and carbon content of samples. (●) persimmon tree pruning branches; (□) HTC1-4, temperature effect; (◇) HTC5-8, time effect; (◯) HTC9,10, solid/liquid effect.

The HHV of pruned persimmon tree branches in this study was determined to be 18.2 MJ/kg, which is comparable to that of other common agricultural biomass materials, such as apple and olive prunings [28,29]. The HHV of samples derived from pruned persimmon tree branches can reach 28.5 MJ/kg, which surpasses that of lignite (typically approximately 25 MJ/kg [30]), contingent upon their carbon content, as illustrated in Figure 5. Excluding the raw material data, the carbon content showed the highest correlation coefficient (0.997) with the HHV based on the least-squares method compared to the O/C (0.976) and H/C (0.919) atomic ratios (Supplementary Material Figure S3). Cellulose extracted from poplar subjected to hydrothermal treatment at 270 °C for 12 h has been reported to exhibit an HHV of approximately 29 MJ/kg [20]. Palm shells were hydrothermally treated at 300 °C for 30 min using citric acid as the solvent to generate an HHV of approximately 23 MJ/kg [23]. The hydrothermal treatment of olive fruit endocarp for less than 10 min at temperatures between 175 and 250 °C resulted in a maximum HHV of approximately 23 MJ/kg [31]. These findings suggest that optimizing hydrothermal conditions according to the feedstock is crucial for achieving a high HHV. Conversely, the pyrolysis of orchards, such as apple trees, has been shown to enhance the HHV from 22 to 30 MJ/kg [32]. This increase in energy density through the hydrothermal treatment of persimmon pruning waste underscores the potential to augment the energy yield. Under these experimental conditions, higher temperatures and longer hydrothermal treatment durations led to an increased energy consumption and decreased solid yield. To improve the fuel properties of the sample converted by hydrothermal treatment, in addition to achieving a higher yield by increasing the solid/liquid ratio, it is essential to optimize both the temperature and duration of the process to minimize energy consumption. The effectiveness of hydrothermal carbonization in improving the fuel properties of non-edible agricultural biomass lays the groundwork for future material use in energy and environmental applications.

## 4. Conclusions

This study investigated the potential of pruned persimmon tree branches as a lignocellulosic feedstock for hydrothermal treatment and conversion into fuel products.

### 4.1. Process Optimization and Enhanced Fuel Properties

Through systematic optimization of the hydrothermal conditions, it was found that treatment at 250 °C for 20 h yielded the converted branches with a high carbon content (69.2%) and a HHV of 28.5 MJ/kg. This value exceeds that of lignite coal, indicating that the investigated system is a competitive solid fuel alternative.

### 4.2. Reaction Mechanisms: Dehydration and Decarboxylation as Key Pathways

Analyses of the structural and compositional changes revealed that dehydration and decarboxylation were the dominant chemical pathways. Hemicellulose rapidly decomposed, cellulose gradually lost crystallinity, and lignin showed partial resistance to degradation. These factors contribute to an increased carbon concentration and improved fuel quality.

## Figures and Tables

**Table 1:** Hydrothermal conditions for pruning the branches of persimmon trees.

Sample Name	Temperature (°C)	Duration (h)	Solid/Liquid Ratio
HTC1	180	20	5
HTC2	205	20	5
HTC3	230	20	5
HTC4	250	20	5
HTC5	230	2	5
HTC6	230	8	5
HTC7	230	16	5
HTC8	230	40	5
HTC9	230	20	2.5
HTC10	230	20	10

**Table 2:** Elemental contents, yields, and CI of the cellulose in the samples.

Sample Name	C	O	H	N	S	Solid Yield (%)	CI (%)
HTC1	52.2	38.3	5.8	0.9	0.0	55.5	68
HTC2	56.1	34.2	5.8	1.0	0.0	51.9	67
HTC3	64.0	24.8	5.3	1.5	0.0	41.5	-
HTC4	69.2	20.4	5.6	1.7	0.0	35.1	-
HTC5	53.6	36.8	5.8	1.1	0.0	56.3	51
HTC6	58.1	31.5	5.6	1.3	0.0	50.0	41
HTC7	61.8	27.3	5.5	1.5	0.0	44.9	29
HTC8	65.1	23.0	5.4	1.7	0.0	36.5	-
HTC9	63.3	26.2	5.3	1.5	0.0	38.0	46
HTC10	63.9	24.6	5.3	1.6	0.5	48.1	-

## Data Availability

The data supporting the findings of this study can be obtained from the corresponding author upon a reasonable request.

## Supplementary Materials

The following supporting information can be downloaded at: https://www.mdpi.com/article/10.3390/ma18153425/s1, Figure S1: Particle size distribution curve of the pulverized pruned persimmon tree branches measured using the laser scattering method (Malvern Mastersizer 3000); Figure S2: FT-IR spectra of HTC9 and 10; Figure S3: Relationship between HHV and the (a) O/C and (b) H/C atomic ratios of the samples; the symbols represent different conditions: (●) persimmon tree pruning branches; (□) HTC1-4, temperature effect; (◇) HTC5-8, time effect; (◯) HTC9,10, solid/liquid effect.
