# Supplementary material for: Hydrothermal Carbonization of Pruned Persimmon Tree Branches: Optimization of Process Conditions for Enhanced Energy Recovery
Source: Materials (Basel). 2025 Jul 22;18(15):3425. doi: 10.3390/ma18153425 (PMC12347359; doi:10.3390/ma18153425)
Supplement: Supplementary file 1 [file materials-18-03425-s001.zip › materials-3723856-supplementary.pptx]

## Slide 1
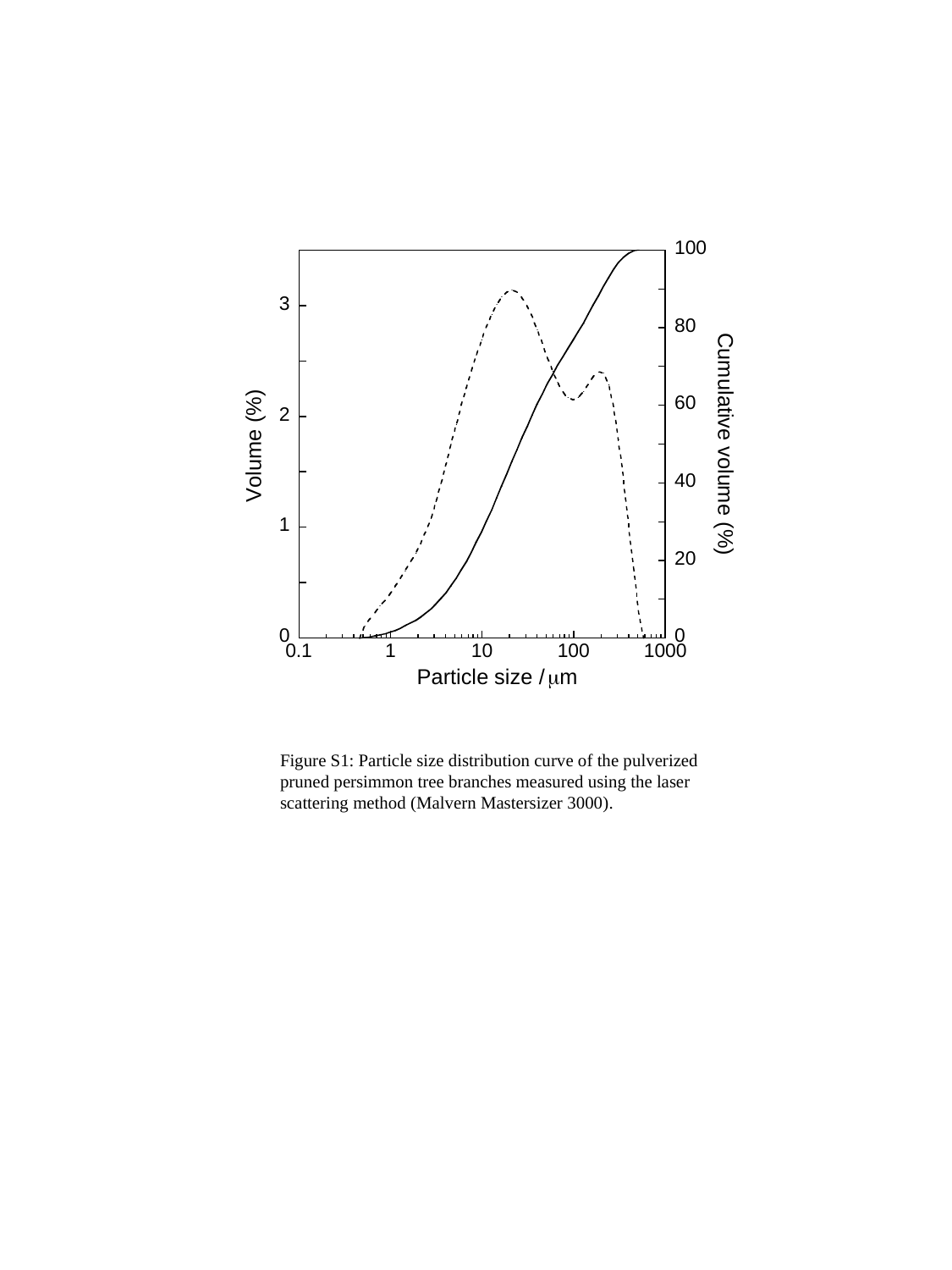

Figure S1: Particle size distribution curve of the pulverized pruned persimmon tree branches measured using the laser scattering method (Malvern Mastersizer 3000).

## Slide 2
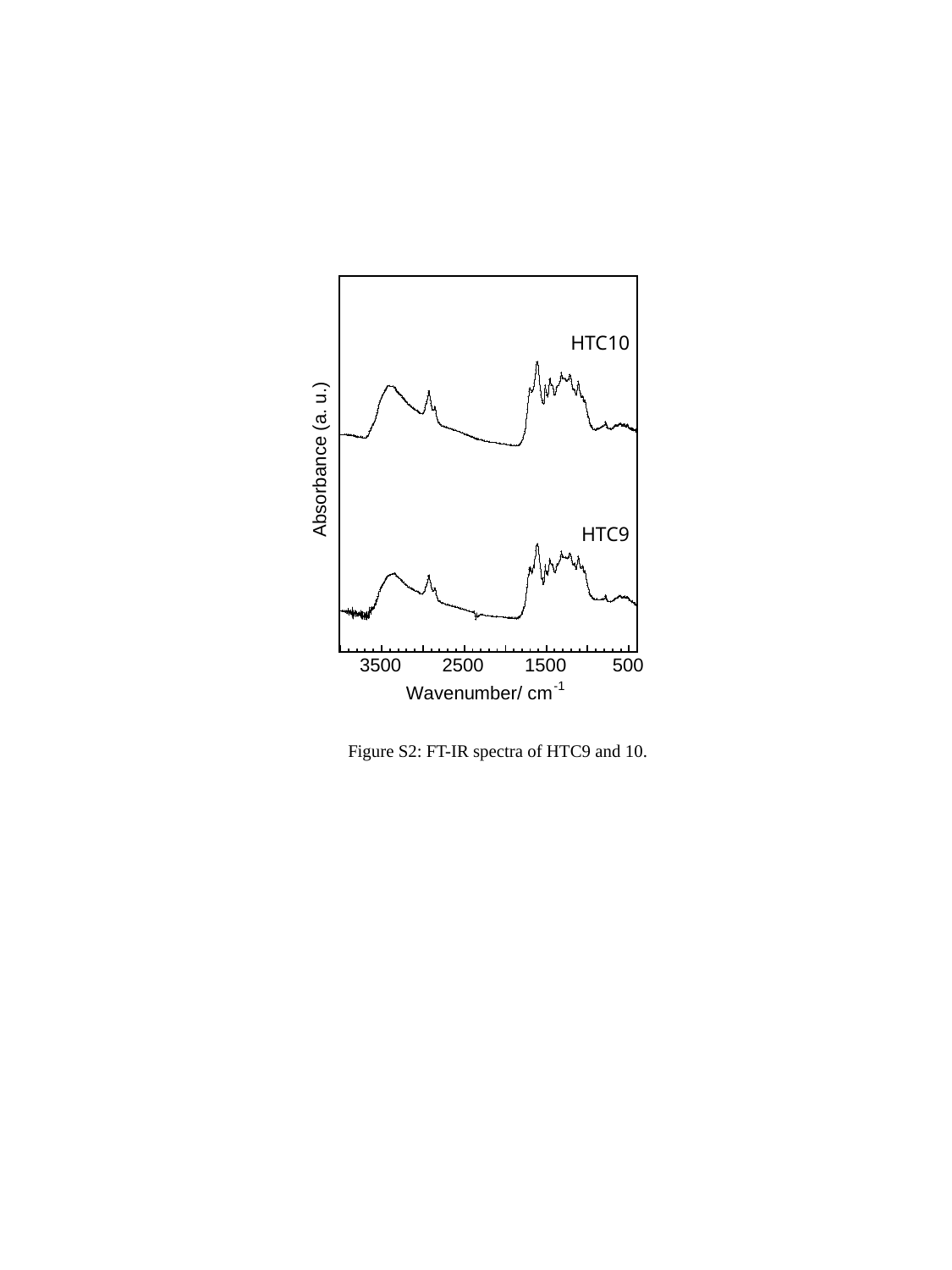

HTC10
HTC9
Figure S2: FT-IR spectra of HTC9 and 10.

## Slide 3
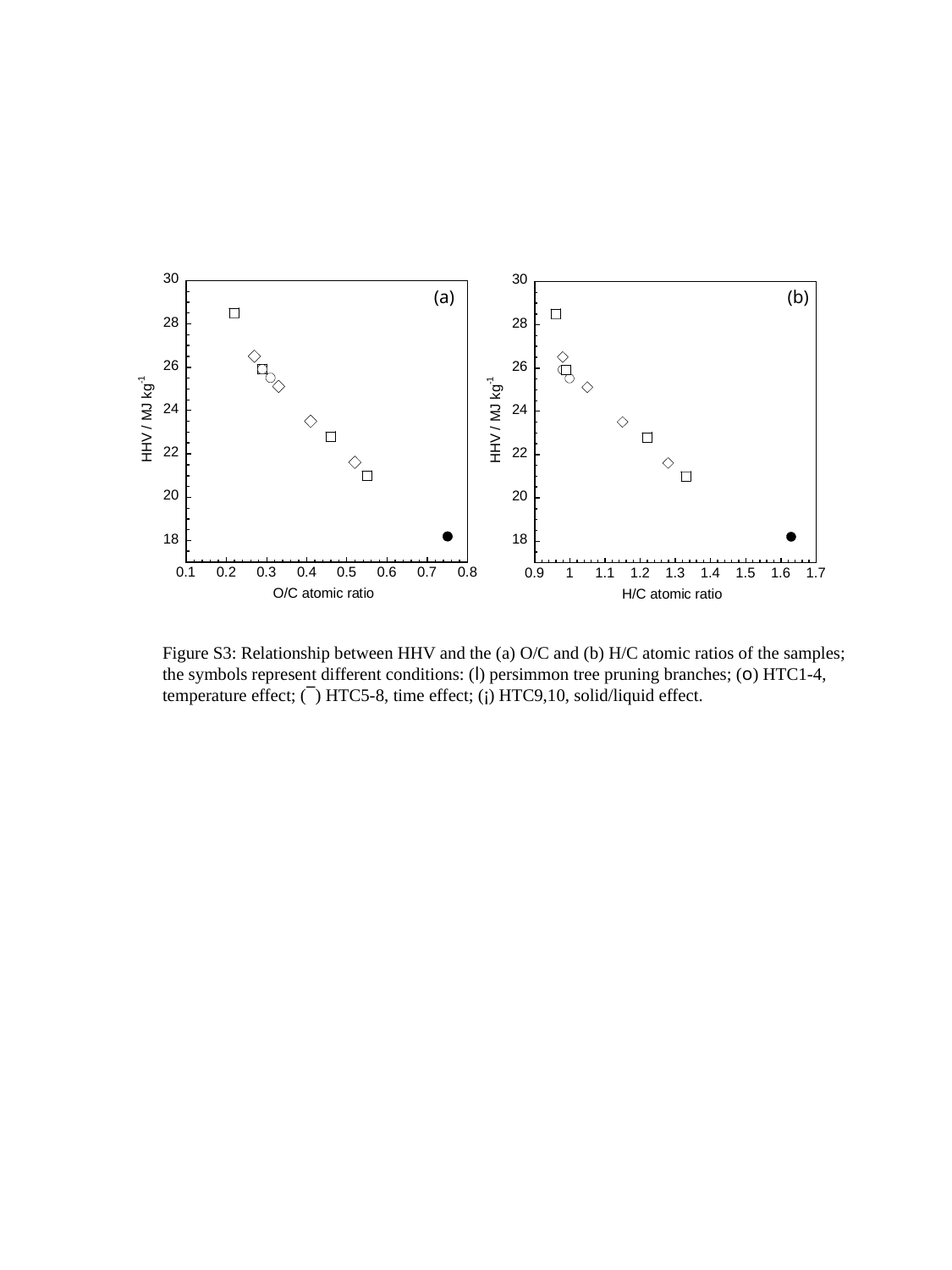

(a)
(b)
Figure S3: Relationship between HHV and the (a) O/C and (b) H/C atomic ratios of the samples; the symbols represent different conditions: (l) persimmon tree pruning branches; (o) HTC1-4, temperature effect; (¯) HTC5-8, time effect; (¡) HTC9,10, solid/liquid effect.
